# Vitamin A Deficiency Induces Congenital Spinal Deformities in Rats

**DOI:** 10.1371/journal.pone.0046565

**Published:** 2012-10-05

**Authors:** Zheng Li, Jianxiong Shen, William Ka Kei Wu, Xiaojuan Wang, Jinqian Liang, Guixing Qiu, Jiaming Liu

**Affiliations:** 1 Department of Orthopaedic Surgery, Peking Union Medical College Hospital, Peking Union Medical College, Beijing, China; 2 Department of Medicine and Therapeutics, Institute of Digestive Diseases, LKS Institute of Health Science, The Chinese University of Hong Kong, Hong Kong, China; IIT Research Institute, United States of America

## Abstract

Most cases of congenital spinal deformities were sporadic and without strong evidence of heritability. The etiology of congenital spinal deformities is still elusive and assumed to be multi-factorial. The current study seeks to elucidate the effect of maternal vitamin A deficiency and the production of congenital spinal deformities in the offsping. Thirty two female rats were randomized into two groups: control group, which was fed a normal diet; vitamin A deficient group, which were given vitamin A-deficient diet from at least 2 weeks before mating till delivery. Three random neonatal rats from each group were killed the next day of parturition. Female rats were fed an AIN-93G diet sufficient in vitamin A to feed the rest of neonates for two weeks until euthanasia. Serum levels of vitamin A were assessed in the adult and filial rats. Anteroposterior (AP) spine radiographs were obtained at week 2 after delivery to evaluate the presence of the skeletal abnormalities especially of spinal deformities. Liver and vertebral body expression of retinaldehyde dehydrogenase (RALDHs) and RARs mRNA was assessed by reverse transcription-real time PCR. VAD neonates displayed many skeletal malformations in the cervical, thoracic, the pelvic and sacral and limbs regions. The incidence of congenital scoliosis was 13.79% (8/58) in the filial rats of vitamin A deficiency group and 0% in the control group. Furthermore, vitamin A deficiency negatively regulate the liver and verterbral body mRNA levels of RALDH1, RALDH2, RALDH3, RAR-α, RAR-β and RAR-γ. Vitamin A deficiency in pregnancy may induce congenital spinal deformities in the postnatal rats. The decreases of RALDHs and RARs mRNA expression induced by vitamin A deprivation suggest that vertebral birth defects may be caused by a defect in RA signaling pathway during somitogenesis.

## Introduction

Congenital spinal deformities are not uncommon with an incidence of approximately 1 per 1,000 live births [1]. Vertebral anomalies may arise from defects in the development of the axial skeleton and are often associated with intraspinal abnormalities (e.g. myelopathy and paraplegia) and other organ defects (e.g. congenital heart disease and kidney defect) [2], [3]. The exact causes of these conditions have not yet been identified. The etiology is thought to be multifactorial, involving both the environmental and genetic factors. Chemical exposure, vitamin B6, and certain drugs have been implicated in the disturbance of vertebral formation [4].

Recent studies have demonstrated that the axial skeleton is formed by a process known as somitogenesis during embryo development [5], [6]. Somites are epithelial blocks, generated in a rhythmic fashion from the paraxial mesoderm, which subsequently differentiate into the vertebrae, ribs, tendons, intercostal and skeletal muscles of the body [7]. Somitogenesis is a precisely organized, multistep process which is believed to be regulated by a molecular oscillator termed the segmentation clock [8]. At least three signaling pathways have been proposed to control the segmentation clock, namely the Notch, Wnt and Fgt pathways [9]. Mutations in components of these signaling pathways have been linked to several malformations, including spondylocostal dysostosis (SCDO), Alagille sydrome (AGS), abnormal vertebral segments (hemivertebrae,wedge vertebrae, block vertebrae), spinal deformities, etc. [10], [11] Recent findings have suggested that disruption of the retinoic acid (RA) pathway may lead to a loss of left-right bilateral symmetry in mouse embryos [12]–[14]. Thus, we hypothesize that RA signaling pathway may also play a role in the development of segmentation clock that regulates the segmental structure of the vertebrate body plan during embroyogenesis.

RA, an active form of vitamin A, plays essential roles in many physiological functions, including vision, immunity, and cell differentiation [15]–[17]. RA signaling is tightly controlled by the opposing actions of retinaldehyde dehydrogenases (RALDHs), which are essential for the generation of embryonic RA from vitamin A, and CYP26 members, which catabolize RA [18]. RA serves a ligand for two families of nuclear receptors (RAR-α, RAR-β, RAR-γ and RXR-α, RXR-β, RXR-γ). Upon ligand binding, these receptors form heterodimers and bind to DNA that harbor the RA response elements (RARES) to directly regulate gene expression at the transcriptional level [19]. Epidemiologic evidence has suggested that vitamin A deficiency (VAD) is not uncommon among pregnant women and children in developing countries [20]. As early as the 1930s, it was realized that maternal VAD results in death of the fetus as well as congenital malformations. The most frequent teratogenic target of VAD was the eye in which VAD-induced ocular defects include coloboma, retinal eversion, penetration of the retina by mesodermal tissue, low insertion of the optic stalk and the cup, and defects in the iris [21]. Abnormalities at lower penetrance were noted in other systems including the genitourinary tract, kidney, diaphrapm, lung, aortic arch, and heart. Nervous system, cardiovascular, and axial patterning defects may be caused by early VAD, whereas a less well-developed nasal region, salivary gland hypoplasia, agenesis of the Harderian glands, hypoplasia of the intestinal villi and a number of skeleton abnormalities arise if VAD occurs at later times [22]. Similar effects have also been observed in embyos of many species of experimental animals with VAD, including monkeys, rabbits, rats, mice, and hamsters [23]. Excess dietary vitamin A, on the other hand, has been shown to cause teratogenesis, but toxicity from food sources is rare. Abnormalities, such as microtia/anotia, micrognathia, cleft palate, conotruncal heart defects and aorticarch abnormalities, thymic defects, retinal or optic-nerve abnormalities and CNS malformations were observed in the neonates of women ingesting therapeutic doses of 13-cis-RA (0.5–1.5 mg/kg) during the first trimester of pregnancy, and these retinoids are, thus, contraindicated for use during pregnancy [21]. RA is a derivative of vitamin A and is required for synchronous left-right development of somites, either maternal VAD during pregnancy or an embryonic defect in metabolism from vitamin A to RA might be associated with increased risk of human vertebral defects.

There has been much research work focusing on the relationship between the skeletal malformation and VAD in embryonic developments. VAD rats exhibit hypoplastic cranial bones, defects of the thyroid, cricoid and tracheal cartilages as well as agenesis of rhe neural arch of cervical vertebrae 1 and ectopic bone in the dorsal regions of C1, malformation of the sternal and pelvic regions [24]. However, the relationship between the congenital spinal deformities (congential scolisosis) and VAD remains poorly understood. The aim of the present study is to examine if severe VAD in pregnant rats could increase the incidence of congenital spinal deformities in filial rats. The effects of VAD on the mRNA expression levels of several components of RA signaling, including RALDH1, RALDH2, RALDH3, RAR-α, RAR-β and RAR-γ, were also investigated.

## Results

### Effect of vitamin A-free diet on maternal rats and embryonic survival

The body weight of VAD and control females before mating were similar (weight gain vs. N = 24 and 8, respectively). The proportion of fertile rats was 45.8% and 75.0% in VAD and control groups, respectively. Data from non-pregnant rats were not included in the results. Meanwhile, maternal rats on VAD diet exhibited an array of VAD symptoms, such as appetite loss, loss of whiskers, patches of fur, ocular porphyrin deposits and depression in response to external stimuli. The number of dead embryos in VAD groups was 5 (45.5%). In contrast, no dead embryos were present in the control group. The same number of neonates was found in VAD and control mothers (10.17±1.47 VS 9.83±0.75, p>0.05; Table 1) There were 61 and 59 filial rats in VAD group and the control group, respectively. Three random neonatal rats from each group were euthanized on the day of birth, the rest of the rats were kept for additional 14 days until they obtained radiography. There was no significant difference in body weight between the mice of VAD group and control group before they obtained radiography (p>0.05; Table 1).

**Table 1:** Characters of filial rats of VAD group and control group.

	VAD group	Control group
Pregnant rate	45.9% (11/24)	75.0% (6/8)
Incidence of embryos dead	45.5% (5/11)	0%
Filial No. of embryonic	10.17±1.47 (61/6)	9.83±0.75 (59/6)
Filial No. of radiography	58	56

### VAD diet reduced serum retinol levels in maternal and neonatal rats

After birth, the mean serum retinol concentration in the adult females on the VAD group was 0.51±0.57 µmol/L, which was significantly lower than that of the control group (2.58±0.12 µmol/L;p<0.01; Table 2). As shown in Table 3, Serum retinol level on day 1 in the offspring in the VAD group was 0.32±0.01 µmol/L, which was significantly lower that those in the control group (1.91±0.09 µmol/L, P<0.01).

**Table 2:** Animal characteristics in the VAD and Control rat groups.

	Maternal rats		Filial rats	
	VAD	Control	VAD	Control
Body weight(g)	304±8.27	306±7.77	29.77±4.11	29.57±5.23
VitA-plasma (µmol)	0.51±0.58**	2.58±0.12	0.32±0.01**	1.91±0.09

** p<0.01, VAD group vs Control group.

**Table 3:** Skeletal abnormalities in VAD and control groups' neonates (% of total).

	VAD group	Control group
Abnormalities in the neural arch of the first cervical vertebra	12.1% (7/58)	0
Malformed sternum (loss of xyphoid)	17.2% (10/58)	0
Dysplasias in the scapula	8.6% (5/58)	0
spinal anomalies in the thoracic regions	13.8% (8/58)	0
Rib fusions	19.0% (11/58)	0
Loss of ribs	6.9% (4/58)	0
deformities of ulna	10.3% (6/58)	0
dysplasia of the ischium	13.8% (8/58)	0
tibia and fibula fusions	6.9% (4/58)	0
Dysplasias in the phalange	12.1% (7/58)	0
Skeltal abnormalities	51.7% (30/58)	0

### VAD induced spinal deformities in neonatal rats

**Figure 1:**
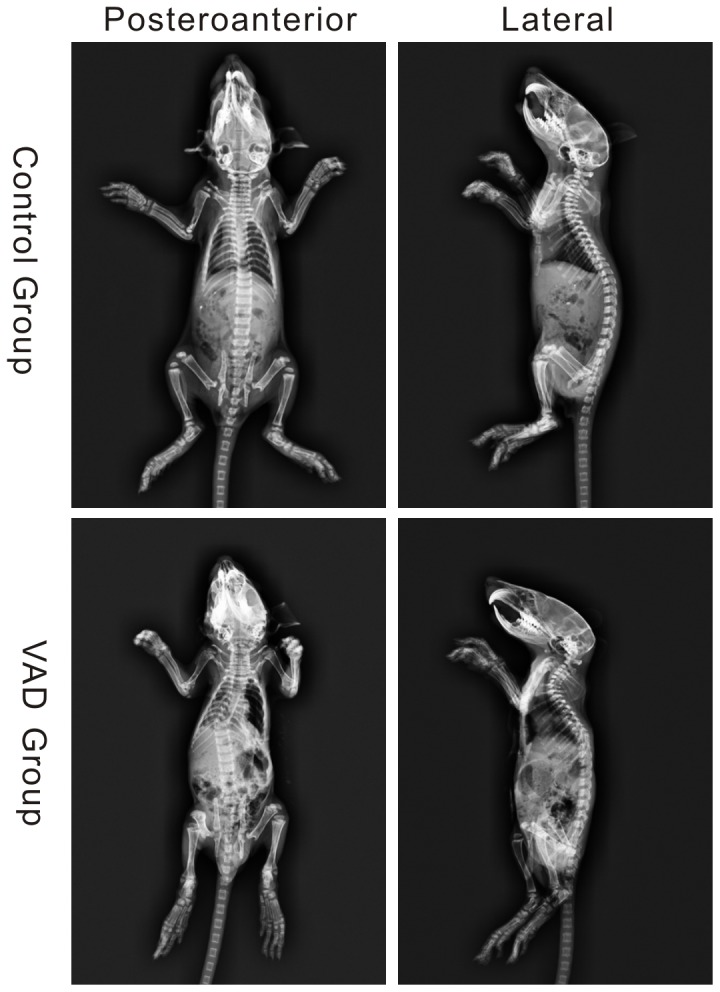
Representative anteropoterior radiographs showing no vertabral anomalies (Control Group) and with congential spinal deformities (VAD Group) original magnification ×2.

The remaining 58 neonates in the VAD group and 56 neonates in the control group were physically examined for spinal deformities. No gross spinal deformities were noticed in all neonatal rats. However, when examined by radiography at post-gestational age of 7 weeks (day 61 after the vaginal plug was documented in the mother), 13.79% (8/58) of the neonatal rats in the VAD group were found to have congenital spinal anomalies in the thoracic regions of the spine (e.g. fused ribs, hemivertebrate, fused vertebrate), many of which were present in multiple regions in the same rats (Fig. 1B). This is strikingly similar to the human clinical situation in which congenital spinal deformities are not noted on gross examination but subsequently discovered on incidental radiographic examination or with the gradual development of spinal deformities as the child grows. The radiographic appearances of the murine spinal deformities are similar to those humans. None in the control group developed any radiographically detectable spinal deformities. Representative radiographs of a normal and a deformed spine from each group are shown in Fig. 1.

### VAD induced skeletal abnormalities in neonatal rats

**Figure 2:**
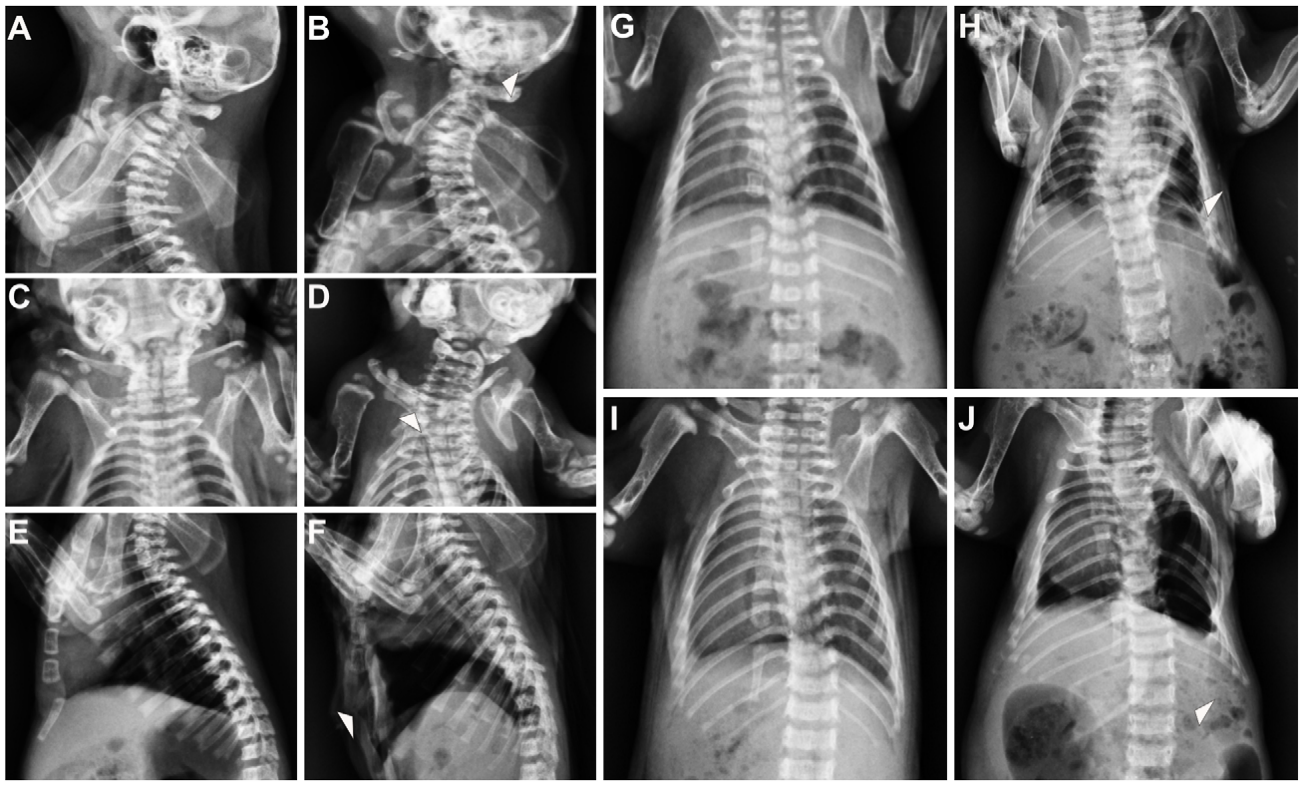
Malformations in the cervical and thoracic regions of VAD neonates. (A) Side view of cervical region showing normal skeletal in a control fetus. (B) VAD fetus showing loss of the neural arch in cervical 1. (C) Ventral view of the scapula region showing normal development in a control fetus. (D) VAD fetus showing dysplasias of the scapula. (E) Side view of the sternal elements showing normal development the manubrium, sternebrae and xyphoid process in a control fetus. (F) VAD fetus showing malformation of the sternal elements as well as loss of xyphoid process. (G) Ventral view of the thoraric region showing normal development in a control fetus. (H) VAD fetus showing anomalies of vertebrae in thoraric region as well as rib fusions. (I) Ventral view of the thoraric region showing normal development in a control fetus. (J) VAD fetus showing loss of rib in vertebral 20.

**Figure 3:**
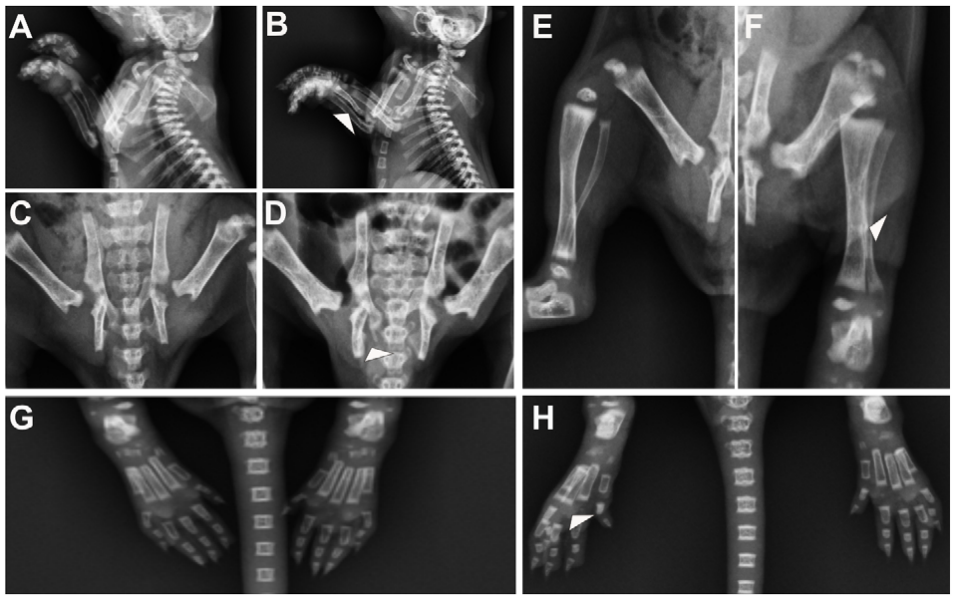
Malformations in the limbs, pelvic and sacral of VAD neonates. (A) Side view of the forelimb region showing normal forelimb development in a control fetus. (B) VAD fetus showing deformities of ulna, at least unilaterally, in the forelimb region. (C) Ventral view of the pelvic region of a control fetus depicting normal development and attachment of the pelvic. (D) VAD fetus showing moderately dysplasia of the ischium. (E) Ventral view of the hindlimb region showing normal hindlimb development in a control fetus. (F) VAD fetus showing malformations of the tibia and fibula as well as tibia and fibula fusion. (G) Ventral view of the ossification region showing normal ossification development in a control fetus. (H) VAD fetus, the ossification of the second phalange was either missing or greatly reduced in size.

Because vitamin A is required for skeletal development during embryo development, we also exmined the other skeletal system of neonatal rats by the same anteroposterior radiography. Various abnormalities were found in the skeletal system of the neonatal rats in the VAD group (Table 3). The cervical vertebrae maintained a normal shape and the first cervical vertebra had a normal neural arch in the neonatal rats from the control group. However, the first cervical vertebra in VAD neonates showed loss of the neural arch in 12.1% (7/58) of VAD neonates (Table 3, Fig. 2.A, B). In addition, 17.2% (10/58) of the VAD neonates showed a partly loss of the sternum (Table 3, Fig. 2.E, F), such as loss of xyphoid process. 6.9% (4/58) of the neonates in VAD group showed a complete loss of the ribs at v20, at least unilaterally (Table 3, Fig. 2.I, J). Rib fusion were also observed in the VAD neonates, with a high prevalence involving the ribs in the region of v16 through 19, where the majority of neonates in VAD group 19.0% (11/58) showed fusions (Table 3, Fig. 2.G, H). In the VAD groups, the development of both the forelimb and hindlimb was abnormal when compared to control neonates. The scapula of the forelimb was abnormal, resulting in misdirection of the acromion process and a failure of articulation with the clavicle and shoulder joint in 8.6% (5/58) of VAD neonates (Table 3, Fig. 2.C, D). In the 10.3% (6/58) of VAD neonates, the ulna was hypoplastic. Malformations were also seen in the pelvic elements of the VAD neonates (Table 3, Fig. 3.A, B). 13.8% (8/58) in the VAD neonates showed dysplasia of the ischium, at least unilaterally (Table 3, Fig. 3.C, D). The hindlimb of 6.9% (4/58) of the VAD neonates was abnormal, showing malformations of the tibia and fibula, such that the tibia and fibula fusion (Table 3, Fig. 3.E, F). Furthermore, the ossification of the second phalange was either missing or greatly reduced in size in the hindlimb of 12.1% (7/58) of VAD neonates (Table 3, Fig. 3.G, H).

### VAD altered the mRNA expression of RALDHs and RARs in liver

**Figure 4:**
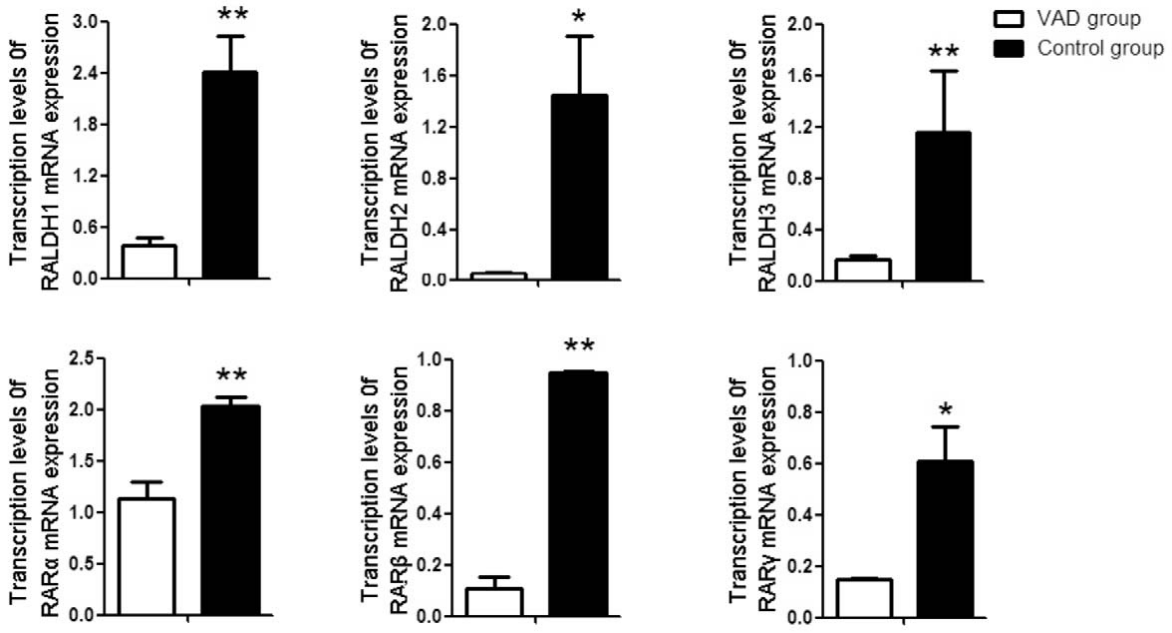
Transcription levels of retinaldehyde dehydrogenase (RALDH1, RALDH2 and RALDH3) and retinoid acid receptors (RARα, RARβ, RARγ) in the liver of control, VAD rat. Bar Mean ±SE of three samples in triplicate. *P<0.05 **P<0.01. VAD group VS Control group.

As shown in Fig. 4, significant decreases in RALDH2 and RAR-γ mRNA expression were observed in livers of VAD rats compared with those of the control rats (p<0.05). Furthermore, the expression of RALDH1, RALDH3, RAR-α, and RAR-β mRNA in livers of the VAD rats was significantly lower than those in the control group as determined by real-time PCR (p<0.01).

### VAD altered the mRNA expression of RALDHs and RARs in vertebral body

**Figure 5:**
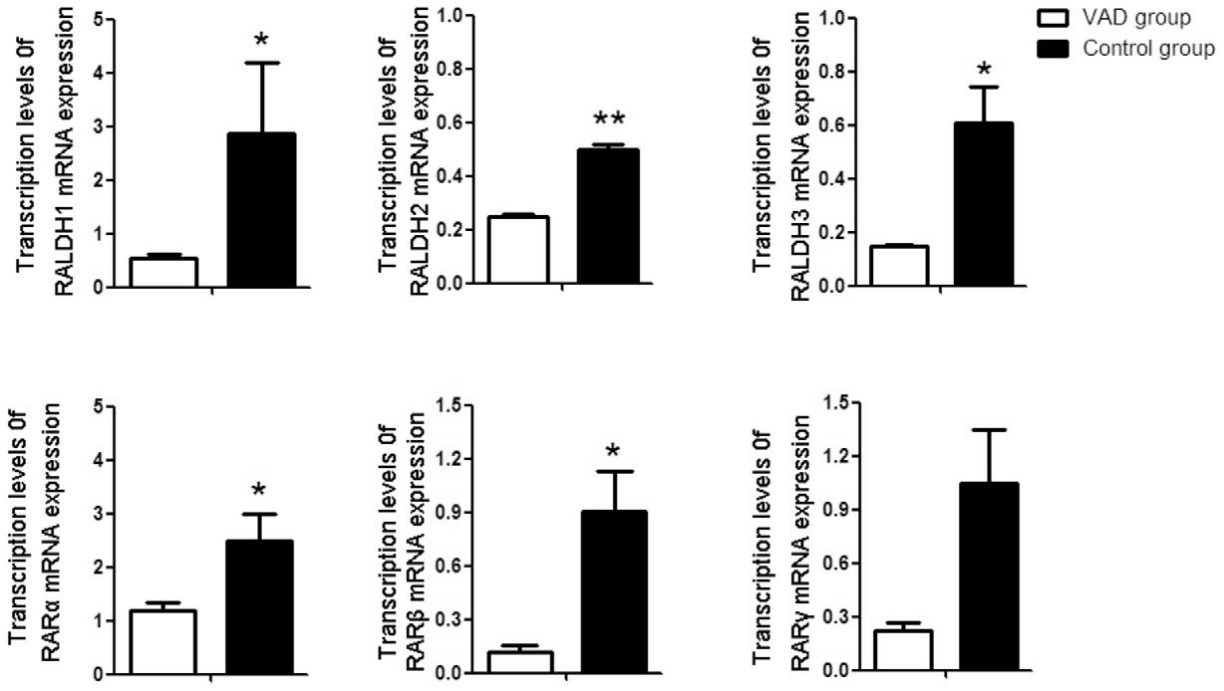
Transcription levels of retinaldehyde dehydrogenase (RALDH1, RALDH2 and RALDH3) and retinoid acid receptors (RARα, RARβ, RARγ) in the vertebral body of control, VAD rat. Bar Mean ±SE of three samples in triplicate. *P<0.05 **P<0.01. VAD group VS Control group.

RALDH1, RALDH2, RALDH3, RAR-α, RAR-β and RAR-γ mRNA were all expressed in the vertebral body of rats. No significant difference was found in the expression level of RAR-γ mRNA in the vertebral body between VAD and control rats (p>0.05). Decreased mRNA levels of RALDH1, RARLD2, RALDH3, RAR-α, and RAR-β were observed in vertebral body of VAD rats as compared with control (p<0.05 for RALDH1, RALDH3, RAR-α, and RAR-β and p<0.01 for RARLD2; Fig. 5)

## Discussion

Congenital spinal deformities are due to anomalous development of the vertebrae including failure of formation and segmentation during embryogenesis, and frequently associated with malformations of other organs (e.g. spinal cord, heart, lung, kidney, etc.) [3]. Patients with congenital spinal deformities normally require observation and may need to undergo surgery in case of curve progression. The etiology and mechanism of congenital vertebral deformities have not yet been fully elucidated. In this regard, various animal models have hinted at the involvement of low oxygen, valproic acid, boric acid, cigarette smoking, fetal alcohol, hyperthermia, and vitamin B6 deficiency in the pathogenesis of congenital spinal deformities, although the exact cellular and molecular mechanisms are not well understood [25]–[28]. The phenomenon that gestational vitamin A deficiency disrupts embryonic development has been known for over 80 years. Teratogenic effects of vitamin A deficiency and excess both involve skeletal morphogenesis, and these abnormalities include hypoplastic cranial bones, defects of the thyroid, cricoid and tracheal cartilages as well as agenesis of the neural arch of cervical vertebrae 1 and ectopic bone in the dorsal regions of C1, malformation of the sternal and pelvic regions [29]. These abnormalities have been widely observed in human and animal models. However, the relationship between the congenital spinal deformities and vitamin A deficiency has been poorly understood. In the present study, we found that VAD is associated with congenital spinal deformities in rats as confirmed by anteroposterior radiography. Our findings support the hypothesis that maternal VAD could induce vertebral anomalies in the offspring. Our study has also observed other skeletal abnormalities include loss of the neural arch of cervical vertebrae 1, malformation of sternum, loss of the ribs, rib fusion, abnormal of forelimb and hindlimb, malformation of the pelvic regions, which have also been observed by others [24], [30]. Another important finding of our study is that VAD repressed the expression RALDHs and RARs, which are important components of RA signaling, in the liver and vertebral body of VAD rats. Although the number of rats in each group is small, the findings are novel and may be relevant to clinical practice, and the difference between the study and control group is very prominent and statistically significant.

Vitamin A is a dietary requirement because of the body's inability to synthesize sufficient quanitities. VAD is still a major public health problem worldwide, which has contributed substantially to the health threat among young children and women of reproductive age in developing coutries. VAD in pregnant women is associated with night blindness, severe anaemia, wasting, malnutrition, and reproductive and infectious morbidity, and increased risk of mortality 1–2 years following delivery [31]. Several previous studies have demonstrated that VAD induces congenital heart, ocular tissues, respiratory, urogenital and circulatory systems, and skeletal deformities in experimental animals including monkeys, rabbits, rats, mice and hamsters [22]. Excess dietary vitamin A, on the other hand, has been shown to also cause teratogenesis. Teratogenic targets of vitamin A excess were the heart, the skull, skeleton, limbs, brain, eyes, CNS, as well as craniofacial structures [32]. However, toxicity of vitamin A excess from food sources is rare. Vitamin A supplies must be regulated to avoid teratogenic consequences from deficiency or over-intake and adequate maternal vitamin A level is important for newborn. To verify the reduction of vitamin A in mice receiving VAD diet, we measured the vitamin A level of mothers before mating and their offspring at postnatal day 1, and confirmed the VAD status during pregnancy in the VAD group as previously described [33]. Previous studies have found that no reduction in food intake and body weight was observed in adult VAD rats [34]. So in our study, there was no difference in body weight between the two groups during the pre-mating and parturition period. Our results show that VAD has little effect on the body weight of the neonate. Although much of skeletal abnormalities were reported as part of the VAD syndrome, interestingly, congenital spinal deformities were not reported in VAD model. The present study now clearly demonstrates that VAD during pregnancy also can induce congenital spinal deformities. As most individuals with congenital spinal deformities have additional congenital abnormalities (spinal cord, heart, lung, kidney, etc.), the underlying defect could be caused by VAD [35], [36]. Here, we demonstrated that maternal VAD induced vertebral anomalies in the offspring are associated with other skeletal deformities and also may be associated with abnormalities in other organs. The skeletal immaturity of the neonatal rats and the breed of Wistar rats and only by X-ray may account for this low incidence rate on skeletal and vertebral anomalies.

How might VAD be acting to interrupt signalling? RA is the active form of vitamin A and plays a crucial role in stimulating nuclear receptor signaling during development. RA synthesis is a two-step process. The first step, oxidation of retinol to retinaldehyde, is catalyzed by several members of the alcohol dehydrogenase family (Adh1, Ad3,and Adh4); the second step, oxidation of retinaldehyde to RA, is catalyzed by three members of the aldehyde dehydrogenase family (RALDH1, RALDH2, RALDH3) and is irreversible [35]. RA synthesis is controlled both spatially and temporally and three RALDHs identified as catalysts for the second step are expressed in dynamic, nonoverlapping spatiotemporal patterns, indicating that this step is tissue- and time-restricted [37]. In vitro studies have shown that RA serves as a ligand for two families of nuclear receptors, namely, RARs and retinoid X receptor (RXRs), both of which bind DNA as heterodimers and directly regulate gene expression [17], [38]. Previous studies have demonstrated that RA is required for embryogenesis and functions through RARs, whereas RXRs are undetectable in mouse embryos [37]. In these studies, RA was shown to be essential for the development of several organs, including the hindbrain, spinal cord, heart, eye, skeleton, forelimb buds, lung, pancreas and genitourinary tract. Moreover, recent studies showed that loss of RA signaling results in left-right asymmetry of somites in mouse, chick, or zebrafish embryos, in which one side has fewer somites than the other [12], [13]. Administration of RA maternally to RA-deficient mouse embryos also restores normal axial turning and normal spinal column development [37]. These findings suggest that RA signaling is implicated in somitogenesis as well as development of other organs and VAD induced reduction RA signaling may be a common cause of congenital spinal deformities and other organ defects.

Another important finding of our study was that VAD caused a decrease in the mRNA levels of RALDH1, RALDH2, RALDH3, RAR-α, RAR-β and RAR-γ in the liver and RALDH1, RALDH2, RALDH3, RAR-αand RAR-β in the vertebral body. Precious studies have demonstrated that RA was synthesized during vertebrate evolution by these three RALDHs (RALDH1, RALDH2 and RALDH3). RA also mediates its effects on embryogenesis exclusively through RARs but not RXRs [19]. Robinson et al [39]using a transcriptomic approach, they compared RA-exposed and nonexposed rat embryos to identify overlapping and nonoverlapping effects of RA on RNA expression, and their finding had indicated that 845 genes were identified to be significantly time-dependent altered by RA in parallel with morphpological and RA induced upregulation of expression of three enzymes, CYP26A1, CYP26B1, and DHRS3, which are known involved in the breakdown of RA. On the other hand, many reports have demonstracted that VAD suppressed RALDHs, RARs and RXRs mRNA expression in many organs of VAD rats [40], [41]. Moreover, the expressions of RAR-α and RAR-β are also severely downregulated in the VAD embryo in the avian [41], [42]. Our results also have observed that VAD suppressed RALDHs and RARs mRNA expression in the liver and vertebral body of VAD neonatal rats at the day of birth. Therefore, our findings suggest that maternal VAD during pregnancy induced congenital spinal deformities in offspring might also be associated with decrease in RA signalling caused by VAD.

A previous study has shown that scoliosis occurs in 74% of birds with vitamin B6 deficiency, while mild VAD did not influence expression of scoliosis. The authors found that the serum retinol levels of birds with mild VAD were 18±6 µg/dl. In their study, the birds were not severely deficient in vitamin A because the investigators used corn oil as the source of dietary lipid [28]. However, in our study, we used the modified AIN-93G diet without any source of vitamin A and the mean serum retinol concentration in the adult females in the VAD group was 0.51±0.57 µmol/L, which was significantly lower than that of the control group (p<0.01).

The findings of the present study may have implications for understanding the etiology and mechanism of human congenital spinal deformities. Given that VAD is still a serious and prevalent public health problem in the world especially among pregnant women and children and dietary vitamin A is only available from limited sources, including vegetables in the form of β-carotene or meat as retinyl esters, VAD may be an important cause of human congenital spinal deformities [43]. Three signaling pathways have been proposed to regulate the segmentation clock, namely the Notch, Wnt, and Fgf pathways. Thus far, mutations in the Notch ligand DLL3, LFNG,MESP2, Notch ligand JAGGED1 and polymorphisms in the Tbx6 gene have been identified to be associated with congenital scoliosis in humans [44]. Strikingly, most of these genes are implicated in the modulation of segmentation clock. Our study shows that RA signaling is implicated in the pathogenesis of human congenital spinal deformities. The role of RA signaling in the regulation of segmentation clock, however, awaits further investigation. Moreover, not all rats in the VAD group develop congenital spinal deformities, suggesting that defects in RA signaling may only confer partial susceptibility.

In conclusion, we here present evidence that congenital spinal deformities may occur in neonatal rats whose mothers were exposed to VAD diet throughout the entire pregnancy. In mothers and neonatal rats, decreases in serum retinoid levels and reduced mRNA expression of RALDHs and RARs in liver and vertebral were noted. These results suggest that vertebral birth defects may be caused by VAD and a defect in RA signaling pathway during somitogenesis. This raises the possibility that an environmentally VAD induced reduction RA signaling paly an important role in the etiology of a wide range of human congenital spinal deformities. To our knowledge, this is the first report presenting the possible association between prenatal VAD and congenital scoliosis. However, large-scale epidemiological studies are still needed to confirm such association in humans. Moreover, effects of partial instead of absolute deprivation of vitamin A on spine malformation should also be examined. Future investigation into the molecular linkage between VAD and somitogenesis will also provide more insight into this phenomenon.

## Methods

### Animal and diets

Thirty two 38-week-old virgin female Wistar, and thirty two 38-week-old male Wistar, pathogen-free rats were obtained from the Experimental Animal Center of Daping Hospital (Third Military Medical University, Chongqing, China). All animals were housed individually in our animal facilities and maintained in an environment under a 12-h light cycle, at a room temperature of 21–23°C with a relative humidity of 60%. Rats were given free access to water and food. The female rats were randomly divided into 2 groups, namely, VAD or control. VAD group (n = 24) were fed a modified AIN-93G diet without any source of vitamin A (Table 4; Research Diets,USA); control group (n = 8) received an AIN-93G diet sufficient in vitamin A (4 retinol equivalents(RE)/g diet). After 1 week of acclimatization, the rats were fed different diets for at least 2 weeks. Plasma levels were analyzed by HPLC in a random sample of animals to verity deficiency (<2 µg of vitamin A per 100 ml, 0.74 µmol/L) as previously descibed [33]. Then the animals were mated with normal males between 6 and 10 p.m. During gestation, the VAD group were fed a modified AIN-93G diet without any source of vitamin A and the control group received an AIN-93G diet sufficient in vitamin A (4 retinol equivalents (RE)/g diet) [45]. Pups born from female rats of the two groups were used for the present study. Three random neonatal rats from each group were killed by cervical dislocation to analyze mRNA expression of liver and vertebral body and to detect serum retinol level on the day of birth. After parturition, the two groups of female rats were fed an AIN-93G diet sufficient in vitamin A (4 retinol equivalents (RE)/g diet) to feed the rest of neonates for two weeks until being killed.

**Table 4:** Formulation of modified AIN-93G growing rodent diet without added vitamin A.

Ingredient	Amount
	gm/kg
casein, lactic, 30 mesh	200
l-cystine	3
corn starch	397.486
maltodextrin 10	132
sucrose	100
cellulose,BW200	50
cottonseed oil	70
t-butylhydroquinone	0.014
mineral mix S10022G	35
vitamin mix S13002	10
choline bitartrate	2.5

### Serum vitamin A measurement

The tail blood of female rats was collected for detecting serum retinol level before mating and after parturition. Six neonatal rats were anesthetized at the day of birth and the whole blood was obtained from the right ventricle of the heart, which was exposed after thoracotomy. Blood samples were collected in EDTA-coated tubes. All serum vitamin A measurements were carried out within 2 weeks after obtaining the samples. The serum was obtained by centrifugation at 1200× g for 15 min at 4°C. To minimize photoisomerization of vitamin A, the plasma was taken under reduced yellow light and frozen in the dark at −80°C until determination of retinol concentrations. Serum vitamin A concentration was measured using high performance liquid chromatography (150×4.6×5 mm; Aglilent Techologies,Santa Clara,CA) as previously described [46]. All procedures were aslo performed in a dark room to protect the serum from light and prevent oxidation of the compounds.

### Radiographic Examination

At week 2 after birth, all the rats had standard anteroposterior (AP) radiographs of the spine under anesthesia for the evaluation for the presence of spinal deformities. The animal use and care protocols were reviewed and approved by the Committee for Animal Experiments of the Third Military Medical University, China.

### RNA extraction and Real-time PCR

Total liver and vertebral body mRNA was obtained on day 1 of the birth from the two groups and was extracted from frozen liver by using TRIzol reagent (Invitrogen, CA, USA) according to the manufacturer's instructions. RNA was isolated with chloroform and isopropanol, washed with ethanol, and dissolved in water. Quantification of RNA was based on spectrophotometric analysis at 260/280 nm with values between 1.8 and 2.0 confirmed the purity of the RNA samples. A 2-µg sample of total RNA was reverse-transcribed with 200 U of MMLV reverse transcriptase (Invitrogen) using Oligo(dT) primers in a 20 µL reaction mixture following the manufactures' instructions. Relative transcript levels of RALDH1, RALDH2, RALDH3, RAR-α, RAR-β and RAR-γ mRNA were determined by real-time PCR using the iQ5 Real-Time PCR Detection System (Bio-Rad, California, USA). The real-time PCR reaction was composed of 1× SYBR Green fluorescent dye (Takara, Dalian, China), 1 µl forward primers (10 µM), 1 µl reverse primers (10 µM), 1× qPCR mix, 1 µl cDNA. The sequences of the specific primers are shown in Table 5. To produce the melting curve, the reactions were subject to one step at 95°C for 30 s followed by 45 cycles of 95°C for 5 s, 60°C for 10 s, and 72°C for 30 s. The relative gene expression was assessed by the ΔΔCt method. GAPDH was used as an internal control.

**Table 5:** Sequence of Primers Used for RT-PCR Detection of Gene Expression.

Gene	Forward primer(5→3)	Reverse primer(5→3)	Amplicon size (bp)	Annealing temperature (°C)
RALDH1	ACTGTGTCATCTGCTCTG	TTACTCTGCTGGCTTCTT	200	60
RALDH2	ACATCAACAAGGCTCTCA	CCAAACTCACCCATTTCTC	139	60
RALDH3	AGAGGGCTGTTCATCAAA	TGCTGTGAGTCCATAGTC	159	60
RARα	AACAACAGCTCAGAACAAC	CGAACTCCACAGTCTTAATG	97	60
RARγ	AGACTTTTCCCTCACTCTG	TTCGCAAACTCCACAATC	235	60
RARβ	CTTGGGCCTCTGGGACAAAT	TGGCGAACTCCACGATCTTAAT	68	60
GAPDH	AACCTGCCAAGTATGATGA	GGAGTTGCTGTTGAAGTC	119	60

### Statistical Analysis

Statistical analyses were performed using SPSS softare version 17.0 (SPSS Inc. Chicago). Data were expressed as means ± S.E.M. Statistical analysis was performed with student's t-test. P values less than 0.05 were considered statistically significant.
